# Retraction: The PAR2 signal peptide prevents premature receptor cleavage and activation

**DOI:** 10.1371/journal.pone.0305363

**Published:** 2024-06-06

**Authors:** Belinda Liu, Grace Lee, Jiejun Wu, Janise Deming, Chester Kuei, Anthony Harrington, Lien Wang, Jennifer Towne, Timothy Lovenberg, Changlu Liu, Siquan Sun

Following the publication of this article [1], the corresponding author contacted PLOS requesting retraction of the article due to concerns with Figs 6 and 7.

Specifically, in a recent attempt to reproduce data presented in Figs 6B–6C and 7B–7C, the authors identified discrepancies between the raw experimental data and the data set used to prepare the two figures.

- In Fig 6B–6C, the conclusion that further deletion of the tethered ligand rescues the functional expression of PAR2 without the signal peptide is not supported by the raw experimental data.
- In Fig 7B–7C, the raw experimental data are inconsistent with the conclusion that the R36A mutation rescues PAR2-SP expression.

The authors stated that Figs 6 and 7 present the main evidence supporting the novel function of PAR2 signal peptide in preventing premature receptor cleavage and activation, whereas data in Figs 1–5 were consistent with the known function of any signal peptide, and data in Figs 8 and 9 were the supporting expression data. Therefore, the authors no longer have confidence in the conclusion of the published article.

In light of the above concerns, the authors retract this article.

All authors agreed with the retraction.
